# Identification of neoepitopes recognized by tumor-infiltrating lymphocytes (TILs) from patients with glioma

**DOI:** 10.18632/oncotarget.24955

**Published:** 2018-04-13

**Authors:** Davide Valentini, Martin Rao, Qingda Meng, Anna von Landenberg, Jiri Bartek, Georges Sinclair, Georgia Paraschoudi, Elke Jäger, Inti Harvey-Peredo, Ernest Dodoo, Markus Maeurer

**Affiliations:** ^1^ Centre for Allogeneic Stem Cell Transplantation (CAST), Karolinska University Hospital Huddinge, Stockholm, Sweden; ^2^ Therapeutic Immunology Unit (TIM), Department of Laboratory Medicine (LABMED), Karolinska Institutet, Stockholm, Sweden; ^3^ Department of Neurosurgery, Copenhagen University Hospital Rigshospitalet, Copenhagen, Denmark; ^4^ Department of Neurosurgery, Karolinska University Hospital, Stockholm, Sweden; ^5^ Krankenhaus Nordwest, Division of Oncology and Hematology, Frankfurt, Germany

**Keywords:** tumor-infiltrating lymphocytes, immunotherapy, neoepitopes, glioblastoma, interferon gamma, Immunology

## Abstract

Neoepitope-specific T-cell responses have been shown to induce durable clinical responses in patients with advanced cancers. We explored the recognition patterns of tumor-infiltrating T lymphocytes (TILs) from patients with glioblastoma multiforme (GBM), the most fatal form of tumors of the central nervous system. Whole-genome sequencing was used for generating DNA sequences representing the entire spectrum of ‘private’ somatic mutations in GBM tumors from five patients, followed by 15-mer peptide prediction and subsequent peptide synthesis. For each mutated peptide sequence, the wildtype sequence was also synthesized and individually co-cultured with autologous GBM TILs, which had been expanded *in vitro* with a combination of interleukin (IL)-2, IL-15 and IL-21. After seven days of culture, interferon gamma (IFN-γ), tumor necrosis factor alpha (TNF-α) and/or IL-17A production was measured by ELISA in culture supernatants, and used as an epitope-specific immune response readout. Mutated peptides that induced a strong cytokine response were considered to contain legitimate neoepitopes. TILs from 5/5 patients with GBM exhibited specific immune reactivity profiles to the nominal target peptides, defined by IFN-γ and/or TNF-α production, as well as IL-17A. Neoepitopes, defined by mutated peptides inducing IFN-γ and/or TNF-α production without or only minimal reactivity to the wildtype sequences, were found for each individual patient. CD8+ TILs dominated the patients’ responses to private neoepitopes. The present study shows that neoepitope-specific TIL reactivity constitutes an important arm of anti-tumor immune responses in patients with GBM, and thus a powerful tool for developing next-generation personalized immunotherapies.

## INTRODUCTION

The identification of T-cell epitopes provided from host-derived targets is crucial in developing personalized treatments for patients with advanced cancer. Epitopes are identified based on the amino acid sequence derived from antigens recognized by a specific population of T cells defined by immune reactivity, i.e. cytokine production, usually measured as antigen-induced interferon gamma (IFN-γ) responses. This process does not only allow to identify new cancer-specific targets, but also to identify T-cell receptors (TCR) that can be used in the development of immunotherapies.

Several approaches of epitope identification are currently in use. Mass spectrometry-based sequencing of peptides eluted from tumor cell-derived human leukocyte antigen (HLA) molecules aims to decipher naturally processed and presented peptides, although there may be limits concerning sensitivity in this approach [1, 2]. Screening of cDNA libraries encoding tumor-associated antigens (TAAs) has been used extensively; this approach is labor-intensive and minimally effective, as larger transcripts and weakly expressed transcripts cannot be cloned easily. Furthermore, this method also eliminates identification of some mutated antigens since it does not cover post-transcriptional modifications of protein antigens [2]. The peptide-based screening approach identifies mutations through whole-exome sequencing followed by *in-silico* analysis, based on an algorithm that predicts the peptide binding capacity of the major histocompatibility complex (MHC)-peptide complex [3, 4]. The drawback of this approach is that the antigen-receptor interaction is based on an algorithm that predicts the MHC binding capacity, and is not thoroughly studied for MHC class II alleles or infrequent HLA alleles [2, 5]. The discovery of epitopes using this approach is only as good as the *in-silico* prediction itself, which is limited for MHC class II alleles and for a number of MHC class I molecules. A novel and revolutionary method is the tandem minigene (TMG) approach, where the patients’ ‘private’ mutations are identified using whole-exome sequencing in order to subsequently construct a personalized library of gene sequences encoding mutated epitopes – or neoepitopes. In the TMG setting, only a small portion of the gene around the mutation is synthesized, and its reaction with autologous T cells is tested to identify whether the predicted neoepitopes are naturally processed and presented to the immune system [6] based on the assumption that the surrogate antigen-presenting cells process and present the neoepitopes in a similar fashion to tumor cells.

In the present study, we have used a combination of whole-exome sequencing and individual peptide responses to screen for immune reactivity among *in vitro*-expanded tumor-infiltrating lymphocytes (TILs) from patients with glioblastoma (GBM) [7], the most lethal form of brain cancer marked by a 5-year survival rate of under 3% [8, 9]. The approach described here allows for direct loading of 15-mer peptides onto MHC class II molecules, or by uptake, processing and subsequent presentation of potential epitopes by MHC class I or MHC class II molecules on surrogate antigen-presenting cells (APCs) to enable T-cell engagement and activation. We report a method that helps the identification of antigenic (neo) epitopes that are presented either directly or indirectly (via processing) to T cells in the tumor microenvironment, a way by which clinically relevant TIL responses can be gauged and tailored for personalized immunotherapy.

## RESULTS

### Clinical characteristics of patients

Five patients were diagnosed with GBM (WHO grade IV tumor of the central nervous system), involving different anatomical compartments of the brain. A description of the clinical characteristic of the patients is provided in Table 1.

**Table 1:** Clinical characteristics of patients

Patient ID	Age	Sex	Diagnosis	Grade	Tumor localization
GBM-A	67	M	GBM	IV	Thalamus
GBM-B	62	F	GBM	IV	Temporal
GBM-C	63	F	GBM	IV	Temporal
GBM-D	65	M	GBM	IV	Frontal
GBM-E	67	F	GBM	IV	Parietal

### TIL reactivity to wildtype and mutated peptides

**Figure 1:**
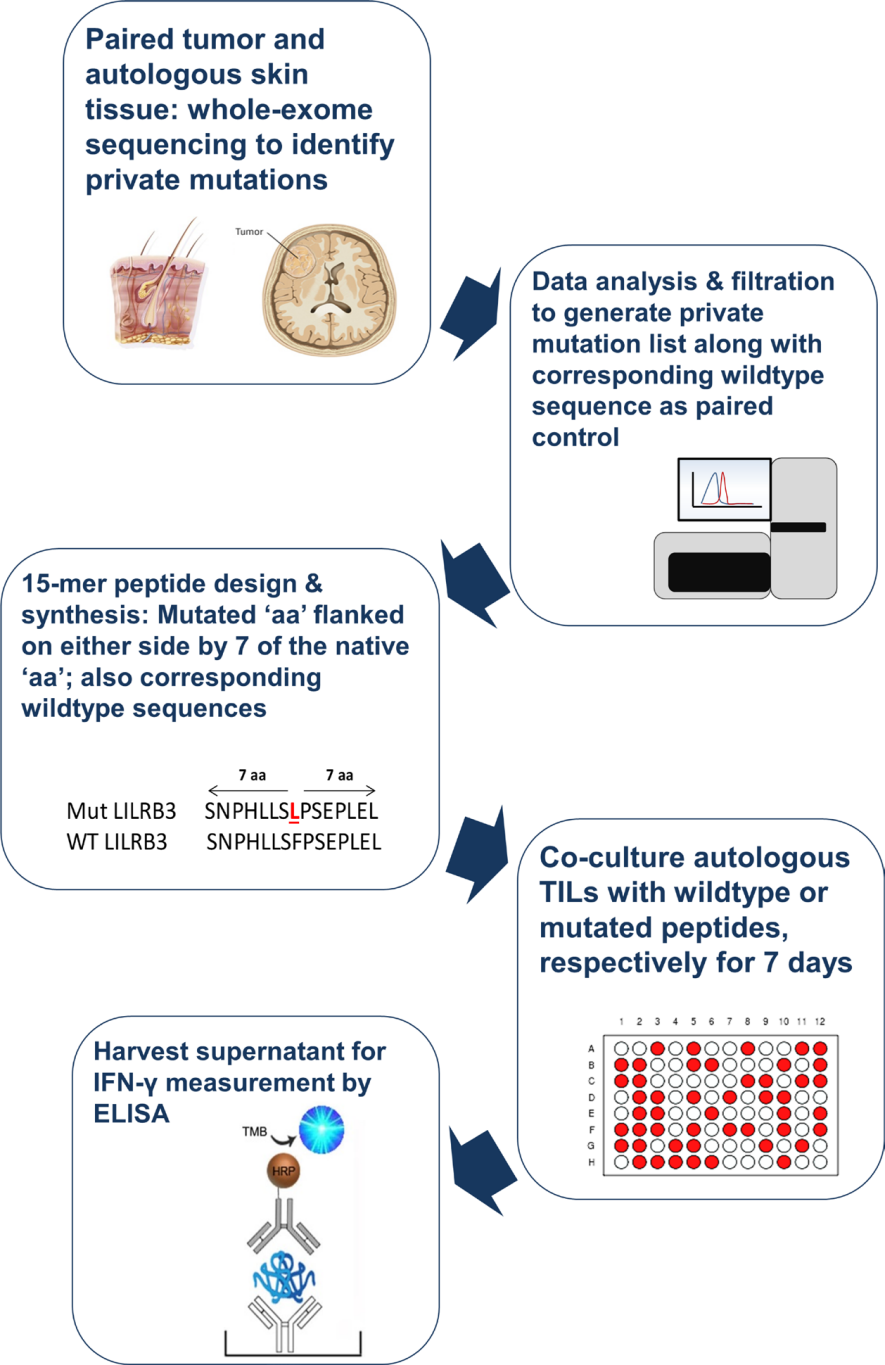
Schematic representation of process workflow Tumor tissue and autologous skin biopsies from patients with GBM (*n* = 5) were submitted for genomic DNA extraction and whole-exome sequencing. The list of private mutations unique to each patient, alongside the corresponding native nucleotide sequence were translated into peptide sequences. 15-mer peptide sequences, with the mutated amino acid in the middle (thus flanked by 7 amino acids on either side) were synthesized. The resulting mutated (mut) peptides as well as the wildtype (wt) counterparts were exposed to autologous tumor-infiltrating lymphocytes over a 7-day period to induce cytokine production, which was then measured using commercially available ELISA kits.

**Figure 2:**
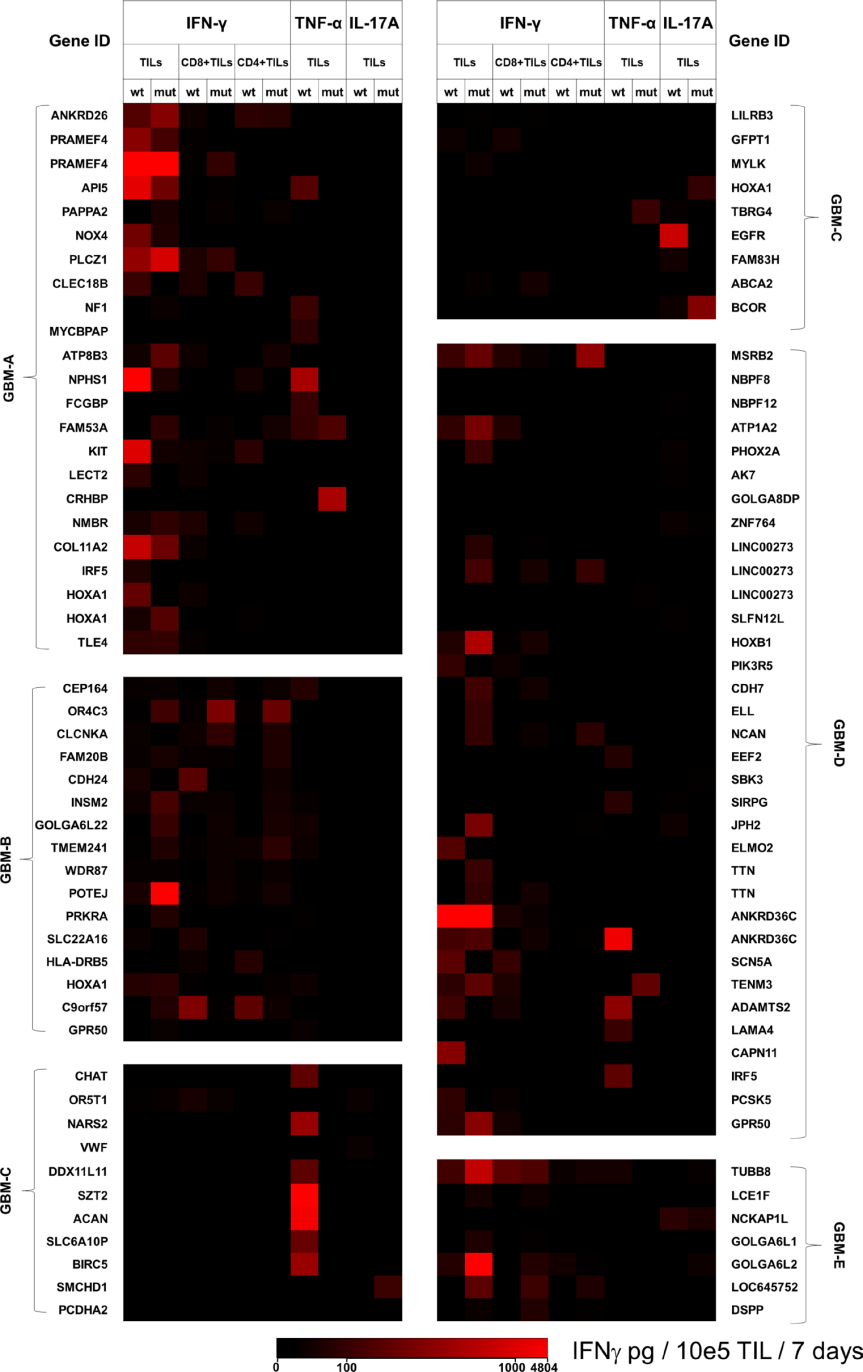
Cytokine production by TILs in response to predicted mutated peptides and corresponding wildtype sequences TILs from five patients with GBM were exposed to mutated peptides predicted to be private tumor-specific epitopes, along with the corresponding wildtype sequences. After seven days of culture, supernatants were harvested for measurement of IFN-γ, TNF-α and IL-17A by sandwich ELISA. Based on the actual concentration of cytokines produced by TILs co-cultured with the individual wildtype or mutated peptides, heat maps were created depicting the peptide-induced responses unique to each patient. A scale ranging from low (black) to high (red) amounts of cytokine produced by total TILs, the CD4+ as well as the CD8+ TIL subsets are shown. Also shown in the scale are cytokine concentration values ranging from 0–4804 pg/10e5 TIL/days. The identical gene name appears several times for some patients due to recognition of different epitopes/peptides belonging to the same gene product. Actual cytokine concentrations are provided in Supplementary Table 1.

A schematic representation of the entire process workflow pertaining to the current study is presented in Figure 1. Immunoreactivity of the TILs from five different patients with GBM to private epitopes (patient-specific peptides generated from the whole-exome sequencing data) were evaluated in TIL cultures. A heat map depicting cytokine responses (IFN-γ, TNF-α and/or IL-17A) following a 7-day *in vitro* stimulation assay with the wildtype as well as mutated peptide sequences was generated for each patient (Figure 2). In Table 2, we show how many mutations from GBM lesions (5 patients) are recognized by TILs based on IFN-γ production. The amount of cytokine produced by TILs, considered as a readout for target-specific immunoreactivity, is presented based on a scale of low to high cytokine concentration in the culture supernatants (Figure 2). A more comprehensive list, detailing individual cytokine concentration values (in pg/10e5 TIL/7 days) per peptide for the five patients with GBM is also presented in Supplementary Table 1. Mutated and wildtype peptides alike were able to activate CD4+ and CD8+ TILs to produce a single cytokine or a combination of the three cytokines measured (IFN-γ, TNF-α and/or IL-17A). We noticed that a positive cytokine response by TILs to the peptides tested (mutated and/or wildtype) was dominated by IFN-γ production. TILs from patients GBM-A and GBM-D appeared to exhibit the strongest overall peptide-driven Th1 immune responses (IFN-γ and/or TNF-α). Several of these peptides (mutated and/or wildtype) also resulted in IL-17A production by TILs. Patients GBM-A and GBM-B displayed only weak to very low IL-17A responses to any of the peptides tested.

**Table 2:** Number of mutations that were recognized by TILs/number of mutations identified by whole DNA exome sequencing

	GBM-A	GBM-B	GBM-C	GBM-D	GBM-E
**TILs**	16/48	17/26	4/92	16/75	9/22
**CD8+TILs**	6/48	14/26	4/92	10/75	7/22
**CD4+TILs**	4/48	16/26	0/92	5/75	6/22

While TILs from patient GBM-B produced measurable amounts of IFN-γ to twelve out of eighteen peptide pairs (mutated and/or wildtype) and did not mount a strong IL-17A response to any of the peptides tested, TILs from patient GBM-E produced substantial amounts of IFN-γ in response to ten out of eleven mutated peptides. Interestingly, TILs from patient GBM-E also produced a combination of IFN-γ and IL-17A in response to three mutated peptides. In terms of peptide-driven TNF-α production by TILs, positive responses were seen mostly with wildtype peptides, with some exceptions (GBM-A = two mutated epitopes, GBM-C = one mutated epitope, GBM-D = four mutated epitope). TILs from patient GBM-C had the weakest IFN-γ response to the peptides tested, but also the strongest TNF-α response to the wildtype peptides, compared to all five patients. TIL from this patient also showed a measurable IL-17A response, particularly to wildtype peptides. Five mutated peptides induced only IL-17A production in TILs from three patients (GBM-A = two peptides; GBM-C = two peptides; GBM-D = one peptide), albeit in the low to medium range (median concentration value = 138.6 pg/ml). None of the peptides tested (mutated and/or wildtype) induced a combination of TNF-α and IL-17A production *in lieu* of IFN-γ by TILs from any of the patients with GBM.

When the individual peptides were tested for activation of (sorted) CD4+ or CD8+ TIL subsets in regard to IFN-γ production, there appeared to be a generally stronger CD8+ rather than CD4+ TIL response to the test candidate peptides from 5/5 patients with GBM (Figure 2/Table 2). CD8+ TILs from patient GBM-A exhibited a broader IFN-γ production in response to wildtype peptides (twelve peptides) as compared to the mutated sequences (six peptides). CD4+ TILs from the same patient had a similar response pattern to wildtype (six peptides) as well as mutated peptide sequences (four peptides). Patient GBM-B also displayed strong CD8+ and CD4+ TIL responses to wildtype peptides, but an equally potent production of IFN-γ was also observed with the mutated sequences. Patient GBM-C only showed IFN-γ responses by CD8+ TILs to two wildtype peptides and four mutated peptides – which appeared to be more focused. While CD8+ TILs from patient GBM-D had comparable responses to the wildtype and mutated peptide sequences, the CD4+ TILs recognized five mutated peptides and none of the wildtype sequences. Last but not least, TILs from patient GBM-E had both CD8+ and CD4+ TILs with a rather highly focused IFN-γ response pattern, recognizing mainly mutated sequences rather than the wildtype peptides.

We also examined in greater detail genes encoding the protein source of the respective peptides tested, in relation to the cytokine responses. The Gene Cards online suite (www.genecards.com) [10] and the UniProt database (www.uniprot.org) [11] were used as a reference. TILs from patient GBM-A showed very strong IFN-γ responses to a mutated peptide and its corresponding wildtype counterpart from the preferentially expressed antigen in melanoma family member 4 (PRAMEF4) protein, which belongs to a group of previously described human melanoma-specific cytotoxic T-lymphocyte antigens with a potential role in retinoic acid signaling [12]. Similarly, TILs from patient GBM-D produced high amounts of IFN-γ in reaction to both a wildtype and mutated peptide pair derived from the ankyrin repeat domain-containing protein 36C (ANKRD36C), which is expressed on immune cells (including lymphocytes) with ion channel-inhibitory properties. Strong IFN-γ production in response to a wildtype peptide of the stem cell factor or Kit, which is also implicated in the regulation of antigen-specific T-cell memory [13, 14], was also seen among TILs from patients GBM-A.

TILs from patient GBM-C showed a strong IL-17A response to a wildtype peptide sequence derived from epidermal growth factor receptor (EGFR), and a strong TNF-α response to a wildtype survivin (BIRC5) peptide. Pertaining to immune responses to proteins associated with the central nervous system, we identified IFN-γ and TNF-α responses to a mutated peptide of teneurin transmembrane protein 3 (TENM3), which is involved in neuronal and potentially visual development [15] (TILs from patient GBM-D). TILs from patients GBM-A and GBM-B had measurable IFN-γ and/or TNF-α reactivity to wildtype as well as mutated peptides from the homeobox protein Hox-A1 (HOXA1), which plays an important role in hindbrain and cardiovascular development [16]. TILs from patient GBM-C had measurable IFN-γ responses to a mutant peptide of the ATP binding cassette subfamily A member 2 (ABCA2), a protein that is strongly expressed in the brain, with involvement in neuronal development [17].

Some striking TIL responses to peptides related to the immune system were also observed. TILs from patients GBM-A and GBM-D showed immune reactivity (IFN-γ and TNF-α production, respectively) to a wildtype peptide from interferon regulatory factor 5 (IRF5), a transcription factor that is involved in the functionality and phenotypic polarization of macrophages [18]. Strong IFN-γ response to a wildtype as well as mutated peptide sequence derived from apoptotic inhibitor 5 (API5) were observed in TIL from patient GBM-A. The abrogation of API5 protein expression in human cervical cancer cells has been implicated in tumor regression and sensitivity to chemotherapy [19]. Patient GBM-C had TILs which responded with TNF-α production to a mutated peptide provided by the transforming growth factor beta regulator 4 (TBRG4), which is involved in cell growth and proliferation via the transforming growth factor beta (TGF-β) signaling pathway. A recent study showed that gene silencing of TBRG4 using short-hairpin RNA structures reduces the viability of the U87MG primary human GBM cell line in culture [20]. In addition, CD8+ TILs also produced IFN-γ in response to a mutated peptide derived from leukocyte immunoglobulin like receptor B3 (LILRB3), which has a role in reducing non-specific inflammation, upregulating the MHC-I pathway and focusing the cellular immune response [21]. In a control experiment, we also observed IFN-γ responses to mutated peptides derived from TBRG4, LILRB3 as well as EGFR among peripheral blood T cells from patient GBM-C, in addition to some unique recognition patterns compared to TILs (Supplementary Table 2).

Mutated peptides that induced an IFN-γ and/or TNF-α response (exclusively or stronger than the corresponding wildtype peptide) were selected and listed in a table (Table 3). This list also includes the mutated peptides that induced IL-17 production, in addition to IFN-γ and/or TNF-α.

**Table 3:** List of mutated peptides with promising neoepitope characteristics

Patient ID	IFN-γ		TNF-α		IFN-γ + TNF-α	
	Sequence	Gene ID	Sequence	Gene ID	Sequence	Gene ID
GBM-A	QVLLEGEHCWLGAKV	PAPPA2	HPLPSAEQYIDFCES	CRHBP		
	HRKSLLLTIFQWKF	NF1				
	ILLSLGF.	ATP8B3				
	HHHHHHHRHPQPATY	HOXA1				
GBM-B	RVLFVVFIYVVTVCG	OR4C3				
	LVVGRFVSLRTEIKP	FAM20B				
	APLSAALKSLKRAAG	INSM2				
	IREQEEMTQEQEEKM	GOLGA6L22				
	PTGDLFSILDFPFLY	TMEM241				
	DIKEKLCFVALDFEQ	POTEJ				
	MSQSRHRL	PRKRA				
	VILFRLLVVILFGRL	C9orf57				
GBM-C	SNPHLLSLPSEPLEL	LILRB3				
	WSIGVICSILVSGLS	MYLK				
	TLVKRPAKPGGPQEP	ABCA2				
GBM-D	PDAVGKCGSAGIKVI	ATP1A2	ADPIPSGRSPGPCGA	LINC00273	GKGVMLAISQGRVQT	TENM3
	EGGPAAPHLGSRTAP	LINC00273				
	NRPTSGPRQRHTRRS	LINC00273				
	DMYGTGQQSLYS	CDH7				
	QSYKNDFSAEYSEYR	ELL				
	ARKAKYNLHATVRYQ	NCAN				
	FARKLKDIHETLGFP	TTN				
	EPDNIKYMISEEKGS	TTN				
	AATSHPKHIKPATSH	GPR50				
GBM-E	SSGGCCGSSSGGCCS	LCE1F				
	REDAGAGEEDVGAGG	GOLGA6L1				
	IREQEEMIREQEAQR	GOLGA6L2				
	PPTWSGRHAPGDRDN	LOC645752				
	QFLIPTSFSVSSNSV	DSPP				

Mutant peptides that induced strong IFN-γ and/or TNF-α responses, including those eliciting a measurable IL-17A response were listed for each patient. Also shown are proteins from which the respective peptide sequences are derived. The gene IDs LINC00273 and TTN appear twice for patient GBM-D due to recognition of different epitopes/peptides derived from the same protein products. The point mutation within the peptide sequence is marked in red (please refer to Supplementary Table 1 for the corresponding wildtype sequences).

## DISCUSSION

The present study describes T-cell epitope mapping that combines whole-exome sequencing, identification of private mutations in a patient's tumor DNA (compared to non-transformed tissue), synthesis of the respective mutated peptide sequence as well as the corresponding wildtype sequence for comparison and finally testing T-cell responses directed against specific mutated and wildtype epitopes found within the peptide sequences.

The detection of an immune response to neoepitopes among cytokine-expanded TILs, in particular CD8+ TILs may reflect (i) existence of T cells in the tumor microenvironment harboring neoepitope-specific TCRs; (ii) neoepitopes that induce a positive T-cell response are in fact naturally presented to the immune system by the corresponding HLA alleles and do not represent simply an artefact; (iii) potential cross-reactivity of neoepitopes with TCRs of other specificities due to molecular mimicry or epitope spreading. It is also possible that naturally processed epitopes within the tumor could be aberrantly processed or trimmed by the endoplasmic reticulum aminopeptidase 1 (ERAP1) protein within the endoplasmic reticulum (ER), leading to degenerate, non-immunogenic MHC-I-compatible sequences [22]. Induction of productive anti-tumor CD8+ TIL responses may therefore be subdued in the tumor microenvironment and mutant epitopes may not bee seen by the cellular immune system, due to differential processing and presentation by MHC molecules. Vice versa, wildtype epitopes may also not be presented and the (point) mutation itself may render the epitope fitting for processing and presentation by the MHC class I – class II pathways. The possibility to revitalize and restore in a robust fashion the neoantigen-specific immune reactivity of TIL populations *in vitro* with IL-2, IL-15 and IL-21 conditioning holds promise for future clinical protocols in immuno-oncology. The use of the TMG method (described in the introductory section) will aid the study of whether wildtype and/or mutant epitopes can be naturally processed and presented to T cells (and TILs in particular), although these experiments are usually performed using B cells or dendritic cells from the respective patient. Furthermore, the presentation of the nominal target antigen (thus, epitopes) by MHC molecules on transformed cells may be substantially different [23, 24].

It is important to note that some mutated peptides derived from GBM tumors are exclusively recognized, yet not their wildtype counterparts. Other patterns of immune reactivity show stronger recognition of the mutated epitope compared to the wildtype peptide sequence. Positive cytokine responses i.e. IFN-γ/TNF-α production, in response to stimulation with mutated peptide sequences indicate that T-cell populations are present within TIL that have the capacity to recognize mutant target epitopes if exclusively the mutant target epitope is recognized (and not the corresponding wildtype epitope), it is highly likely that TIL have been expanded and stimulated by naturally presented T-cell neoepitopes Such uniquely recognized mutated epitopes may present excellent candidates for additional selection and expansion of tumor-specific T cells, in particular TILs directed against neoepitopes that are preferably or exclusively generated by mutational events (mutations, splicing or stop codons) [25]. This is particularly important for GBM, given the high degree of genetic heterogeneity within the tumor before and after chemotherapy [26, 27]. However, T-cell responses within TIL are usually polyclonal and the ‘net-response’ of cytokine production may therefore stem from several T-cell clones directed against the same nominal target. A detailed examination of TIL reactivity of mutant versus wildtype epitope sequences would therefore require T-cell cloning and a more detailed molecular composition analysis of TIL populations directed against defined tumor epitopes.

Anti-tumor responses are in effect productive and very focused autoimmune responses that lead to beneficial clinical outcomes. In this regard, some TIL subpopulations (in particular neoepitope-specific CD8+ T cells) may be polyfunctional, since they are capable of cytokine production as well as cytotoxic activity [25, 28]. The nature of the epitope itself, its capacity to bind to the groove of the MHC molecule and the strength of recognition by the TCR (affinity) are collectively reflected in the cytokine release assays reported here. Production of pro-inflammatory cytokines such as TNF-α, IFN-γ, type 1 interferons (IFN-α/β) and IL-18 may increase antigen turnover and promote further activation of a wider range of bystander immune effector cells [29, 30]. Thus, an optimal immune response elicited by only a small population of neoepitope-specific TILs may lead to the mobilization of other T cells as well as B cells – which may potentially be reflected in neoepitope-specific antibody responses.

TILs have also been proposed as a reliable biomarker of the efficacy of pharmacological agents, as shown in the example of breast cancer treatment [31, 32]. In addition, neoepitope-specific T cells have been identified as an important target of immune checkpoint blockade therapy of patients with metastatic melanoma or non-small cell lung cancer i.e. treatment with anti-programmed cell death-1 (PD-1) and/or anti-cytotoxic T lymphocyte-associated antigen-4 (CTLA-4) monoclonal antibodies [33]. A more clonal neoepitope response, hence a stricter TCR repertoire of T cells, appears to promise a higher likelihood of eliciting clinically beneficial effects in patients with metastatic cancer. Patients with GBM, in particular those who have recurrent disease, are likely to develop further mutations in the tumor after temozolomide therapy [27]. Thus, a mutational analysis of the tumor following chemotherapy may uncover neoepitopes, which could elicit T-cell reactivity and be used for adjunct immunotherapy. The delivery of tumor-reactive T cells could either be systemic, or intra-cranial, as reported using chimeric antigen receptor (CAR) transgenic T cells to patients with GBM [34, 35], the same route of delivery maybe considered for neoepitope-specific T cells after surgery [36].

We noticed that patients GBM-C (770 days) and GBM-E (582 days) had a generally better survival profile post-surgery compared to the other three patients (between 300 and 550 days). TIL from both patients exhibited focused IFN-γ responses to neoepitopes; patient GBM-C had an exclusively CD8+ TIL-centric reactivity while patient GBM-E exhibited a combination of CD4+ and CD8+ TIL responses to neoepitopes. In addition, TILs from patient GBM-E recognized mutated peptides involved in the TGF-β signaling pathway, which has a poor prognosis in human GBM [20, 27], the ABCA2 cellular protein that is highly expressed in the brain (recognized by CD8+ TILs), as well as LILRB3, which is implicated in fine-tuning the MHC-I-dependent immune response (recognized by CD8+ TILs) [21]. This observation suggests a possible correlation between focused neoepitope-specific T-cell responses and a survival benefit among patients with GBM, as previously reported for patients with metastatic melanoma or non-small cell lung cancer [33].

The existence of measurable and potentially durable immune responses directed against a distinct repertoire of private neoantigens in patients with advanced cancers has clinically relevant implications also for the development of personalized cancer vaccines [37]. Conjugate vaccine constructs encompassing various patient-specific neoepitopes can be formulated for delivery alongside a potent adjuvant [37, 38]. Vaccines may then be administered systemically, or via the intra-arterial as well as intra-cranial (intra-tumoral) routes, depending on feasibility of surgical intervention and the vaccine formulation, particularly since peripheral blood mononuclear cells (PBMCs) are also able to react (without *ex vivo* stimulation) to mutant epitopes, that are either unique or shared with neoepitopes recognized by TILs (Supplementary Table 2).

We also measured IL-17A TIL responses in this study, although the role of this pro-inflammatory cytokine in cancer has been controversial. Studies have shown that IL-17 may have a beneficial effect on tumor growth and disease progression, while some preclinical research efforts indicate that IFN-γ-dependent IL-17 production by T cells can lead to elimination of tumors *in vivo* and establish tumor-specific immune responses [39, 40]. Furthermore, IL-17 has also been attributed to induction of tumor-specific cytotoxic T-lymphocytes in the tumor microenvironment, in part due to increased MHC class I and II antigen processing and presentation [41, 42]. In a comprehensive review by Punt *et al*., a higher frequency of IL-17+ T cells in tumor tissue as well as peripheral blood from patients with various types of solid cancers appeared to correlate with improved survival [43]. This evidence therefore prompted us to include IL-17-inducing mutated peptides in the selection of potential neoepitopes associated with GBM, with the condition that IFN-γ responses were also observed. It is therefore plausible that selective IL-17 responses to exclusively mutated but not the wildtype epitopes may give rise to clinically beneficial responses i.e. tumor regression. This can be tested in future clinical trials, where mutation – specific TIL responses are mapped for IFN-γ, as well as for IL-17 production. Neoepitope-induced immunological tolerance, owing to enhanced regulatory T-cell (Treg) activity can be a limiting factor in clinical efficacy after reinfusion of TILs [44]. This effect may however by counteracted by the presence of IL-21 in the T-cell expansion medium, since effector CD4+ and CD8+ T cells have been shown to develop resistance to CD4+CD25+ Treg-mediated immune suppression following exposure to IL-21 [45]. We have also previously shown that GBM TILs cultured with IL-2, IL-15 and IL-21 have very low frequencies of FoxP3+ CD4+ CD25^hi^ Tregs after the expansion phase [7]. The fact that the expansion protocol used in this study does not induce Tregs is highly desirable for clinical use, since the presence of Tregs in the infusion product may dampen the expected anti-tumor effect of TILs in the patients. TIL infusion products with very low or no Tregs therefore represent enhanced quality with greater chances of mediating productive immune responses and positive clinical outcomes.

In conclusion, neoepitope identification using the method described in this study allows the characterization of TIL populations that are responsive to autologous cancer cells based on cytokine production (IFN-γ alone or in combination with other cytokines, e.g. IL-17). Furthermore, by combining the readout obtained by peptide-based screening and the TMG approach, it is possible to narrow down the epitope repertoire for selective and specific design of TCRs for developing efficacious personalized treatments for patients with advanced cancers. The fast screening approach will also allow for the better selection of TILs to be reinfused into the patient as adjunctive immunotherapy.

## MATERIALS AND METHODS

### Patient characteristics

GBM tumor samples (*n* = five patients; designated as GBM-A to GBM-E) were used to generate TILs, which was approved by the Regional Ethical Review Board (Regionala etikprövningsnämnden) at Karolinska Institutet, Sweden (EPN: 2013/576-31 & 2013/977-31/1). A brief description of the patients’ clinical characteristics is provided in Table 1.

### Generation of TILs from GBM tumors

TILs from five patients with GBM were isolated and propagated *in vitro* (in the presence of IL-2, IL-15 and IL-21) as previously described [7, 46]. Briefly, tumor tissue was cut into 1–2 mm^3^ pieces with a sterile scalpel, washed twice with ice-cold PBS and cultured in 24-well plates in T-cell medium (Cellgro GMP-grade serum-free medium; CellGenix, Catalogue Number: 20801-0500) with 5% pooled human AB serum (Innovative Research, Catalogue Number: IPLA-SERAB-14668), supplemented with recombinant human (rh) IL-2 (1000IU/ml) (Prospec, Catalogue Number: cyt-209-b), rhIL-15 (10 ng/ml) (Prospec, Catalogue Number: cyt-230-b) and rhIL-21 (10 ng/ml) (Prospec, Catalogue Number: cyt-408-b). Medium was changed as necessary. Feeder cells, consisting of allogeneic PBMCs which were irradiated at 55 Gy, were added at a ratio of 1 (feeder cells):10 (TILs) on day 7 of culture. Further expansion of the TILs was performed in G-Rex flasks (Wilson Wolf, Catalogue Number: 800400S) with 30 ng OKT3/mL (Miltenyi Biotec, Cat #: 170-076-124) and irradiated allogeneic feeder cells at one feeder cell per five TILs.

### DNA isolation, whole-genome sequencing and mutanome analysis

Genomic DNA purification, library construction, exome capture of approximately 20,000 coding genes, and next generation whole-exome sequencing (WES) of tumor and normal samples isolated from PBMCs were performed using standard operating procedures as previously described by Jones *et al.* [47]. In brief, genomic DNA from tumor and normal samples were fragmented, purified and used for constructing an Illumina DNA library (original method by Illumina, San Diego, CA, USA; modifications described in detail by Jones *et al.* [47]). Exonic regions were captured in solution using the Agilent SureSelect Human All Exon 50 Mb kit Version 3 according to the manufacturer's instructions (Agilent, Catalogue number: G3370A). Paired-end sequencing, resulting in 100 bases from each end of the fragments, was performed using a HiSeq 2000 Genome Analyzer (Illumina, Catalogue number: SY-401-1001). Sequence data were mapped to the reference human genome sequence and sequence alterations were determined by comparison of over 50 million bases of tumor and DNA from non-malignant lesions. Bases of sequence data were obtained for each sample, with a high fraction from the captured coding regions. Over 43 million bases of target DNA were analyzed in the tumor and normal samples, and an average of 42 to 51 reads were obtained at each base in the normal and tumor DNA samples. The tags were aligned to the human genome reference sequence (hg18) using the Eland algorithm of CASAVA 1.6 software (Illumina, San Diego, CA, USA). The chastity filter of the BaseCall software of Illumina was used to select sequence reads for subsequent analysis. The ELANDv2 algorithm of CASAVA 1.6 software (Illumina, San Diego, CA, USA) was then a applied to identify point mutations and small insertions, deletions or stop codons. Mutation polymorphisms recorded in the Single Nucleotide Polymorphism Database (dbSNP) were removed from the analysis. Potential somatic mutations were filtered as previously described [47]. Only non-synonymous single and dinucleotide substitutions, respectively were listed in an Excel spreadsheet. Alternatively spliced products or stop codons may result in epitopes that are shorter than the 15-mer standard peptides used for screening of immune cell reactivity and were therefore omitted.

### Neoepitope synthesis and immune reactivity evaluation in TILs

After identification of mutations through WES and *in-silico* analysis of the sequencing data, 15-mer peptides were constructed by placing the mutation at the center of the 15-amino acid sequence (customized order from Peptide & Elephants, Catalogue Number: A15-554LB). The corresponding wildtype epitope to each mutated sequence was also synthesized for comparison of immune cell reactivity. TILs (1.0 × 10^5^ cells) from patients with GBM were cultured in 200 μl of T-cell medium with 1 μg/ml of the individual wildtype or mutated peptide in round-bottom 96-well microtiter plates. Negative controls contained assay medium alone while the anti-human CD3 antibody (OKT3, 30 ng/mL) was used as the positive control of maximal TCR stimulation. Cells were incubated for 7 days in a CO_2_ incubator at 37°C, after which supernatants were harvested and tested for IFN-γ production using a standard sandwich enzyme-linked immunosorbent assay (ELISA) kit (Mabtech, Stockholm, Sweden). Values from the negative control (medium) were subtracted from antigen (peptide)-specific responses and the data reported to reflect the IFN-γ production (in pg/7 days/1.0 × 10^5^ TILs representing the net IFN-γ production from CD4+ or CD8+ T-cell populations. Cultures of CD4+ and CD8+ TILs with the individual peptides were performed after separation of the T-cell subsets from total TIL cultures using the MACS anti-human CD4+ (Miltenyi Biotec, Catalogue Number: 130-045-101) and anti-human CD8+ (Miltenyi Biotec, Catalogue Number: 130-045-201) microbeads as well as elution columns (Miltenyi Biotec, Catalogue Number:130-042-401).

### Isolation of and immune reactivity evaluation in PBMCs

PBMCs were isolated from heparinized blood or blood products collected from apheresis process over a ficoll hypaque gradient. Similar to the *in vitro* stimulation of TILs, (unseparated) PBMCs (1.0 × 10^4^/well) from patients GBM-C and GBM-E were co-cultured with the wildtype and mutated peptides for seven days at 37°C and with 5% CO_2_. Cell culture supernatants were harvested at the end of the incubation period for IFN-γ detection by sandwich ELISA.

### ELISA for cytokine detection

ELISA was used for detecting the production of IFN-γ (Mabtech, Catalogue Number: 3420-1H-20), TNF-α (Mabtech, Catalogue Number: 3510-1H-20) or IL-17A (Mabtech, Catalogue Number: 3520-1H-20) in cell culture supernatants according to the manufacturer's instructions.

## SUPPLEMENTARY MATERIALS TABLES





## Abbreviations

ANKRD36C: ankyrin repeat domain-containing protein 36C
ABCA2: ATP Binding Cassette Subfamily A Member 2
APCs: antigen-presenting cells
API5: apoptotic inhibitor 5
EGFR: epidermal growth factor receptor
ELISA: enzyme-linked immunosorbent assay
GBM: glioblastoma (multiforme)
GBM-A to GBM-E: patients A to E with glioblastoma
HLA: human leukocyte antigen
HOXA1: homeobox protein Hox-A1
IFN-γ: interferon gamma
IL: interleukin
IRF5: interferon regulatory factor 5
LILRB3: leukocyte immunoglobulin like receptor B3
MHC: major histocompatibility complex
PBMCs: peripheral blood mononuclear cells
PRAMEF4: preferentially expressed antigen in melanoma family member 4
TBRG4: transforming growth factor beta regulator 4
TCR: T-cell receptor
TENM3: teneurin transmembrane protein 3
TILs: tumor-infiltrating lymphocytes
TMG: tandem minigene
TNF-α: tumor necrosis factor
Treg: regulatory T cell
