# Correction: Identification of neoepitopes recognized by tumor-infiltrating lymphocytes (TILs) from patients with glioma

**DOI:** 10.18632/oncotarget.26447

**Published:** 2018-12-04

**Authors:** Davide Valentini, Martin Rao, Qingda Meng, Anna von Landenberg, Jiri Bartek, Georges Sinclair, Georgia Paraschoudi, Elke Jäger, Inti Harvey-Peredo, Ernest Dodoo, Markus Maeurer

**Affiliations:** ^1^ Centre for Allogeneic Stem Cell Transplantation (CAST), Karolinska University Hospital Huddinge, Stockholm, Sweden; ^2^ Therapeutic Immunology Unit (TIM), Department of Laboratory Medicine (LABMED), Karolinska Institutet, Stockholm, Sweden; ^3^ Department of Neurosurgery, Copenhagen University Hospital Rigshospitalet, Copenhagen, Denmark; ^4^ Department of Neurosurgery, Karolinska University Hospital, Stockholm, Sweden; ^5^ Krankenhaus Nordwest, Division of Oncology and Hematology, Frankfurt, Germany

**This article has been corrected:** Please note the following IP filing: IP has been filed for the cytokine cocktail IL-2, IL-15 and IL-21 used for T-cell expansion.

Original article: Oncotarget. 2018; 9:19469-19480. https://doi.org/10.18632/oncotarget.24955

