# Supplementary material for: Identification of neoepitopes recognized by tumor-infiltrating lymphocytes (TILs) from patients with glioma
Source: Oncotarget. 2018 Apr 13;9(28):19469–80. doi: 10.18632/oncotarget.24955 (PMC5929402; doi:10.18632/oncotarget.24955)
Supplement: Supplementary file 1 [file oncotarget-09-19469-s001.pdf]

Identification of neoepitopes recognized by tumor-infiltrating lymphocytes (TILs) from patients with glioma

SUPPLEMENTARY MATERIALS

Supplementary Table 1: List of predicted mutated epitopes and the corresponding wildtype sequence for each patient based on whole-exome sequencing data, as well as the cytokine responses of TILs. Cytokine production is provided in pg/10^5 TILs/7 days corrected by spontaneous cytokine production. See Supplementary_Table_1

Supplementary Table 2: IFN-γ production by peripheral blood T cells from patient GBM-C to mutant and wildtype peptides

ID	Patient code	Wildtype sequence	IFN-γ Wildtype (PBMCs)	IFN-γ Mutant (PBMCs)	Mutated sequence	Gene
1	GBM-C	QTHTEPTGDSKSIKA	503.84		QTHTEPTVDSKSIKA	MKI67
2	GBM-C	PKMLVNFLAKNKSIS	141.67	240.2	PKMLVNFVAKNKSIS	OR5T1
3	GBM-C	CGNVRVNAIYGLIVA	1174.31		CGNVRVNTIYGLIVA	OR52N1
4	GBM-C	GMGCPANRIHTYVYE	34.95		GMGCPANQIHTYVYE	OOSP2
5	GBM-C	FFATVECVLLAAMAY	79.99	291.84	FFATVECILLAAMAY	OR5P2
6	GBM-C	EQEQSSKRLSYQGQS		172.36	EQEQSSKCLSYQGQS	C1orf116
7	GBM-C	GTTRLLSVLFM	55.96	180.01	GTTRLLSALFM	CD55
8	GBM-C	AKTKASPAGKLEARA		111.65	AKTKASPVGKLEARA	FICD
9	GBM-C	TDRALQNKGISAFLV		79.39	TDRALQNQGISAFLV	ACADS
10	GBM-C	DTPSIEKRFAYSFLQ		737.33	DTPSIEKLFAYSFLQ	RYR2
11	GBM-C	DQCSCCSPTRTEPMQ	647.17	194.59	DQCSCCSLTRTEPMQ	VWF
12	GBM-C	ESVESRVLPGPRHRH	308.58		ESVESRVMPGPRHRH	DDX11L11
13	GBM-C	TTDSHIAEEKEDVKR		466.4	TTDSHIAKEKEDVKR	CCDC168
14	GBM-C	DDIACMIGYRPCPWM	491.89		DDIACMIRYRPCPWM	SLC6A10P
15	GBM-C	CNGTWSEDVGNHTKF	143.15		CNGTWSENVGNHTKF	LEPR
16	GBM-C	NRREAEERFNKQSAS	346.22		NRREAEEWFNKQSAS	KRT24
17	GBM-C	FIHCPTENEPDLAQC	909.36	77.89	FIHCPTEKTSQTWPS	BIRC5
18	GBM-C	IVLILTSAILLGSRV		80.35	IVLILTSTILLGSRV	DHRS7C
19	GBM-C	DRMPGKTEPETAGPN		75.42	DRMPGKTDPETAGPN	RASAL3
20	GBM-C	SNPHLLSHPSEPLEL	134.79	382.94	SNPHLLSLPSEPLEL	LILRB3
21	GBM-C	SNPHLLSHPSEPLEL		34.5	SNPHLLSYPSEPLEL	LILRB3
22	GBM-C	YSLWEHSTKNHLQLE		427.08	YSLWEHSMKNHLQLE	APOB
23	GBM-C	RRFVSQETGNLYIAK	147.22		RRFVSQEMGNLYIAK	CNTN6
24	GBM-C	LVTETSSVSTGHATP		72.13	LVTETSSASTGHATP	MUC4
25	GBM-C	DPCSLPLDEGSCTAY		429.75	DPCSLPLGEGSCTAY	COL7A1
26	GBM-C	QVLHLLGPKLEADLE		38.58	QVLHLLGHKLEADLE	QARS
27	GBM-C	QPGSTHTTAFPDSTT		329.66	QPGSTHTRAFPDSTT	MUC12
28	GBM-C	HHHHHHHRHPQPATY		160.85	HHHHHHHHHPQPATY	HOXA1
29	GBM-C	LAFLAESCATLSQEQ		495.61	LAFLAESYATLSQEQ	TBRG4
30	GBM-C	LENLQIIRGNMYYEN		114.62	LENLQIIKGNMYYEN	EGFR
31	GBM-C	ASVDNPHVCRLLGIC		459.85	ASVDNPHMCRLLGIC	EGFR
32	GBM-C	GARGFFQARHLEMDA		136.1	GARGFFQVRHLEMDA	FAM83H
33	GBM-C	EFYYCTEDVLRMGGD		161.97	EFYYCTEVVLRMGGD	ENTPD4
34	GBM-C	TKVESLHEQHSGLKQ	81.13	175.68	TKVESLHAQHSGLKQ	PTPN3
35	GBM-C	VWYSILKDTKITCEE		579.42	VWYSILKGTKITCEE	ASTN2
36	GBM-C	LHILTTLQEPHDKHL	510.13	774.76	LHILTTLEEPHDKHL	TSC1
37	GBM-C	TLVKRPAEPGGPQEP	560.09	370.28	TLVKRPAKPGGPQEP	ABCA2
38	GBM-C	KEYHFISTEEMTRNI	225.72	266.65	KEYHFISMEEMTRNI	MPP1
39	GBM-C	TLRVDRKRKVSGDSS	11.92		TLRVDRKHKVSGDSS	BCOR
40	GBM-C	PLSQESEVEEPLSQE	518.55	235.92	PLSQESEMEEPLSQE	VCX3A
41	GBM-E	IREQEEMLREQEAQR	183.97	1015.07	IREQEEMIREQEAQR	GOLGA6L2

PBMCs from patient GBM-C were exposed to the mutated or wildtype-peptides for a period of seven days. Cell culture supernatants were then collected for IFN-γ measurement by sandwich ELISA. Shown is the actual cytokine values in pg/10^4 PBMCs, corrected by spontaneous cytokine production.
