# Supplementary material for: Identification of neoepitopes recognized by tumor-infiltrating lymphocytes (TILs) from patients with glioma
Source: Oncotarget. 2018 Apr 13;9(28):19469–80. doi: 10.18632/oncotarget.24955 (PMC5929402; doi:10.18632/oncotarget.24955)
Supplement: Supplementary file 2 [file oncotarget-09-19469-s002.doc]

**Supplementary Table 1**

| **ID** | **Patient code** | **Wildtype sequence** | **IFN-γ Wildtype (TILs)** | **IFN-γ Mutant (TILs)** | **IFN-γ Wildtype (CD8+TILs)** | **IFN-γ Mutant (CD8+TILs)** | **IFN-γ Wildtype (CD4+TILs)** | **IFN-γ Mutant (CD4+TILs)** | **TNF-α Wildtype** | **TNF-α Mutant** | **IL-17A Wildtype** | **IL-17A Mutant** | **Mutated sequence** | **Gene ID** |
| --- | --- | --- | --- | --- | --- | --- | --- | --- | --- | --- | --- | --- | --- | --- |
| **GBM-A** | |  |  |  |  |  |  |  |  |  |  |  |  |  |
| **1** | **GBM-A** | **ENFPQKYVDPLAGAA** | 315.48 | 502.08 | 59.14 |  | 170.28 | 149.46 |  |  |  |  | **ENFPQKYIDPLAGAA** | **ANKRD26** |
| **2** | **GBM-A** | **LKNLCVELYPAPRES** | 520.13 | 257.23 | 16.66 |  |  |  |  |  |  |  | **LKNLCVEVYPAPRES** | **PRAMEF4** |
| **3** | **GBM-A** | **KRNKKPVEDCPRMRG** | >961.80 | >961.80 | 20.68 | 194.18 |  |  |  |  |  |  | **KRNKKPVQDCPRMRG** | **PRAMEF4** |
| **4** | **GBM-A** | **KGGTKEKRLAAQFIP** | 865.07 | 415.6 |  | 19.06 |  |  | 324.05 |  |  |  | **KGGTKEKQLAAQFIP** | **API5** |
| **5** | **GBM-A** | **QVLLEGERCWLGAKV** |  | 94.94 |  | 26.67 |  | 37.16 |  |  |  |  | **QVLLEGEHCWLGAKV** | **PAPPA2** |
| **6** | **GBM-A** | **RDEDPRKLLFTTVPG** | 425.59 | 117.54 |  |  |  |  |  |  |  |  | **RDEDPRKVLFTTVPG** | **NOX4** |
| **7** | **GBM-A** | **SALVKGCRCLEIDCW** | 554.81 | 807.54 | 109.65 | 202.98 |  |  |  |  |  |  | **SALVKGCHCLEIDCW** | **PLCZ1** |
| **8** | **GBM-A** | **TPSLASGLWRTLQVG** | 211.86 |  | 107.93 |  | 212.72 |  |  |  |  |  | **TPSLASGPWRTLQVG** | **CLEC18B** |
| **9** | **GBM-A** | **HRKSLLLTDISMENV** |  | 41.44 |  |  |  |  | 224.62 |  |  |  | **HRKSLLLTIFQWKF** | **NF1** |
| **10** | **GBM-A** | **FYFDNREGVILPGEI** |  |  |  |  |  |  | 165.37 |  |  |  | **FYFDNRESVILPGEI** | **MYCBPAP** |
| **11** | **GBM-A** | **ILLSLGFYAIMTTTT** | 61.61 | 344.69 | 56.84 |  |  | 75.83 |  |  |  |  | **ILLSLGF** | **ATP8B3** |
| **12** | **GBM-A** | **ETVSSFYRLNVLYRP** | 956.01 | 109.88 |  |  | 79.76 |  | 626.81 |  |  |  | **ETVSSFYCLNVLYRP** | **NPHS1** |
| **13** | **GBM-A** | **SAGCIPSDKCPPELE** |  |  |  |  |  |  | 210.79 |  |  |  | **SAGCIPSEKCPPELE** | **FCGBP** |
| **14** | **GBM-A** | **SSASGGFVDSSEGSA** |  | 173.3 |  | 24.5 |  | 72.3 | 191.42 | 292.31 |  |  | **SSASGGFMDSSEGSA** | **FAM53A** |
| **15** | **GBM-A** | **IIVMILTYKYLQKPM** | 831.69 | 70.46 | 53.4 | 29.94 | 159.74 |  |  |  |  |  | **IIVMILT** | **KIT** |
| **16** | **GBM-A** | **NAINNGVRISGRGFC** | 161.08 |  | 51.68 |  |  |  |  |  |  |  | **NAINNGV** | **LECT2** |
| **17** | **GBM-A** | **HPLPSAERYIDFCES** |  |  |  |  |  |  |  | 637.65 |  |  | **HPLPSAEQYIDFCES** | **CRHBP** |
| **18** | **GBM-A** | **NEALHFICDPDKREV** | 94.25 | 174.22 | 112.52 |  | 62.56 |  |  |  |  |  | **NEALHFIRDPDKREV** | **NMBR** |
| **19** | **GBM-A** | **PPGPLGKRGPAGSPG** | 744.59 | 403.4 | 40.2 |  |  |  |  |  |  |  | **PPGPLGK** | **COL11A2** |
| **20** | **GBM-A** | **EDVKWPPTLQPPTLR** | 110.79 |  |  |  |  |  |  |  |  |  | **EDVKWPPTLQPPTLQ** | **IRF5** |
| **21** | **GBM-A** | **HHHHHHHRHPQPATY** | 371.76 |  | 51.68 |  |  |  |  |  |  |  | **HHHHHHHHHPQPATY** | **HOXA1** |
| **22** | **GBM-A** | **HHHHHHHHRHPQPAT** | 86.26 | 309.38 |  |  | 20.39 |  |  |  |  |  | **HHHHHHHRHPQPATY** | **HOXA1** |
| **23** | **GBM-A** | **QVIPFLSQEQQLQAQ** | 177.77 | 194.07 | 25.85 |  |  |  |  |  |  |  | **QVIPFLSEQQLQAQH** | **TLE4** |
| **GBM-B** | |  |  |  |  |  |  |  |  |  |  |  |  |  |
| **24** | **GBM-B** | **RDPKSTPTPTYYGSL** | 47.99 | 61.66 |  | 124.46 |  | 84.48 | 250.75 |  |  |  | **RDPKSTPPPTYYGSL** | **CEP164** |
| **25** | **GBM-B** | **RVLFVVFLLIYVVTV** | 16.75 | 436.11 | 53.65 | 875.36 | 41.59 | 721.71 |  |  |  |  | **RVLFVVFIYVVTVCG** | **OR4C3** |
| **26** | **GBM-B** | **LDIKNFGAKVVGLSC** | 66.41 | 10.78 | 102.47 | 330.5 |  | 214.48 |  |  |  |  | **LDIKNFGTKVVGLSC** | **CLCNKA** |
| **27** | **GBM-B** | **LVVGRFVNLRTEIKP** | 62.02 | 164.08 | 59.99 | 51.58 |  | 221.78 |  |  |  |  | **LVVGRFVSLRTEIKP** | **FAM20B** |
| **28** | **GBM-B** | **NTLIDPAYLRRGPFK** | 154.76 | 16.84 | 607.8 | 10.43 |  | 125.07 |  |  |  |  | **NTLIDPA** | **CDH24** |
| **29** | **GBM-B** | **APLSAALQSLKRAAG** | 86.47 | 481.84 | 72.66 | 80.75 |  | 145.57 | 42.24 |  |  |  | **APLSAALKSLKRAAG** | **INSM2** |
| **30** | **GBM-B** | **IREQEEMMQEQEEKM** |  | 376.95 |  | 107.46 |  | 167.72 | 126.6 |  |  |  | **IREQEEMTQEQEEKM** | **GOLGA6L22** |
| **31** | **GBM-B** | **PTGDLFSVLDFPFLY** | 22.26 | 215.73 | 36.42 | 91.41 | 92.96 | 315.41 | 87.09 |  |  |  | **PTGDLFSILDFPFLY** | **TMEM241** |
| **32** | **GBM-B** | **REEEEVREEEEERKE** | 50.99 | 55.57 | 4.56 | 90.53 | 26.53 | 71.67 |  |  |  |  | **REEEEVRKEEEERKE** | **WDR87** |
| **33** | **GBM-B** | **DIKEKLCYVALDFEQ** | 170.58 | >1776.13 | 32.79 | 99.43 | 37.34 | 133.62 |  |  |  |  | **DIKEKLCFVALDFEQ** | **POTEJ** |
| **34** | **GBM-B** | **MSQSRHRA** |  | 233.73 |  |  |  | 149.31 | 22.07 |  |  |  | **MSQSRHRL** | **PRKRA** |
| **35** | **GBM-B** | **YILGVVTAMVGKFAI** | 69.71 | 11.91 | 209.7 | 4.34 |  | 17.99 |  |  |  |  | **YILGVVTDMVGKFAI** | **SLC22A16** |
| **36** | **GBM-B** | **FLEDRRAAVDTYCRH** | 6.89 |  | 92.54 |  | 252.86 |  |  |  |  |  | **FLEDRRAEVDTYCRH** | **HLA-DRB5** |
| **37** | **GBM-B** | **PHHHHHHHHRHPQPA** | 263.32 | 298.16 | 14.59 |  | 11.14 | 43.66 | 103.41 |  |  |  | **PHHHHHHHRHPQPAT** | **HOXA1** |
| **38** | **GBM-B** | **VILFRLLGVILFGRL** | 3.58 | 227.17 | 890.36 | 29.63 | 664.35 | 107.29 |  |  |  |  | **VILFRLLVVILFGRL** | **C9orf57** |
| **39** | **GBM-B** | **AATSHPKPTTGHIKP** |  | 68.42 |  |  |  |  | 56.42 |  |  |  | **AATSHPKHIKPATSH** | **GPR50** |
| **GBM-C** | |  |  |  |  |  |  |  |  |  |  |  |  |  |
| **40** | **GBM-C** | **ALQLLHGGGYSKNGA** |  |  |  |  |  |  | 1785.49 |  |  |  | **ALQLLHGRGYSKNGA** | **CHAT** |
| **41** | **GBM-C** | **PKMLVNFLAKNKSIS** | 61.41 | 140.87 | 417.37 | 179.79 |  |  |  |  | 222.07 |  | **PKMLVNFVAKNKSIS** | **OR5T1** |
| **42** | **GBM-C** | **QWYLDLRRFGSVPHG** |  |  |  |  |  |  | 2837.51 |  |  |  | **QWYLDLRQFGSVPHG** | **NARS2** |
| **43** | **GBM-C** | **DQCSCCSPTRTEPMQ** |  |  |  |  |  |  |  |  | 175.34 |  | **DQCSCCSLTRTEPMQ** | **VWF** |
| **44** | **GBM-C** | **ESVESRVLPGPRHRH** |  |  |  |  |  |  | 1747.21 |  |  |  | **ESVESRVMPGPRHRH** | **DDX11L11** |
| **45** | **GBM-C** | **ELERQMKMENLFVTW** |  |  |  |  |  |  | 4804.21 |  |  |  | **ELERQMKIENLFVTW** | **SZT2** |
| **46** | **GBM-C** | **HLSSIVTPEEQEFVN** |  |  |  |  |  |  | 4580.32 |  |  |  | **HLSSIVTSEEQEFVN** | **ACAN** |
| **47** | **GBM-C** | **DDIACMIGYRPCPWM** |  |  |  |  |  |  | 1959.19 |  |  |  | **DDIACMIRYRPCPWM** | **SLC6A10P** |
| **48** | **GBM-C** | **GTEEDGGGVGHRTVY** |  |  |  |  |  |  |  |  |  | 1113.44 | **GTEEDGGSVGHRTVY** | **SMCHD1** |
| **49** | **GBM-C** | **SNPHLLSHPSEPLEL** |  | 74.33 |  | 59.7 |  |  |  |  |  |  | **SNPHLLSYPSEPLEL** | **LILRB3** |
| **50** | **GBM-C** | **DGRLSIHRIKRTAGD** | 234.43 |  | 413.09 |  |  |  |  |  |  |  | **DGRLSIHQIKRTAGD** | **GFPT1** |
| **51** | **GBM-C** | **WSIGVICYILVSGLS** |  | 247.19 |  | 34.68 |  |  |  |  |  |  | **WSIGVICSILVSGLS** | **MYLK** |
| **52** | **GBM-C** | **HHHHHHHHRHPQPAT** |  |  |  |  |  |  |  |  |  | 956.11 | **HHHHHHHRHPQPATY** | **HOXA1** |
| **53** | **GBM-C** | **LAFLAESCATLSQEQ** |  |  |  |  |  |  |  | 1065.73 | 148.54 |  | **LAFLAESYATLSQEQ** | **TBRG4** |
| **54** | **GBM-C** | **LENLQIIRGNMYYEN** |  |  |  |  |  |  |  |  | 3778.01 |  | **LENLQIIKGNMYYEN** | **EGFR** |
| **55** | **GBM-C** | **GARGFFQARHLEMDA** |  |  |  |  |  |  |  |  | 351.3 |  | **GARGFFQVRHLEMDA** | **FAM83H** |
| **56** | **GBM-C** | **TLVKRPAEPGGPQEP** |  | 144.31 |  | 390.29 |  |  |  |  |  |  | **TLVKRPAKPGGPQEP** | **ABCA2** |
| **57** | **GBM-C** | **TLRVDRKRKVSGDSS** |  |  |  |  |  |  |  |  | 285.19 | 2450.25 | **TLRVDRKHKVSGDSS** | **BCOR** |
| **GBM-D** | |  |  |  |  |  |  |  |  |  |  |  |  |  |
| **58** | **GBM-D** | **WPSFSEAHGTSGSDE** | 428.21 | 747.66 | 261.4 | 80.56 |  | 1054.84 |  |  |  |  | **WPSFSEARGTSGSDE** | **MSRB2** |
| **59** | **GBM-D** | **NLKEKCFLTQLAGFL** |  |  |  |  |  |  |  |  | 9.24 |  | **NLKEKCFVTQLAGFL** | **NBPF8** |
| **60** | **GBM-D** | **LLTPDEPDKSQGQDL** |  |  |  |  |  |  |  |  | 18.28 | 3.1 | **LLTPDEPEKSQGQDL** | **NBPF12** |
| **61** | **GBM-D** | **PDAVGKCRSAGIKVI** | 353.94 | 865.82 | 261.4 | 10.56 |  |  |  |  |  |  | **PDAVGKCGSAGIKVI** | **ATP1A2** |
| **62** | **GBM-D** | **ARCSSEDDDSKESTC** |  | 412.16 |  |  |  |  |  |  | 47.28 |  | **ARCSSEDSKESTCSP** | **PHOX2A** |
| **63** | **GBM-D** | **RWEEWNKRLEEVKRE** |  |  |  |  |  |  |  |  | 30.69 |  | **RWEEWNK** | **AK7** |
| **64** | **GBM-D** | **PGEGHGGEHLDSEGE** |  |  |  |  |  |  |  |  | 7.41 |  | **PGEGHGGDHLDSEGE** | **GOLGA8DP** |
| **65** | **GBM-D** | **PSDLRRHVRTHTGEK** |  |  |  |  |  |  |  |  | 81.47 | 24.61 | **PSDLRRHMRTHTGEK** | **ZNF764** |
| **66** | **GBM-D** | **EGGPAAPRLGSRTAP** |  | 288.05 |  | 19.5 |  |  |  |  |  |  | **EGGPAAPHLGSRTAP** | **LINC00273** |
| **67** | **GBM-D** | **NRPTSGPWQRHTRRS** |  | 495.14 |  | 169.74 |  | 395.59 |  |  |  |  | **NRPTSGPRQRHTRRS** | **LINC00273** |
| **68** | **GBM-D** | **ADPIPSGLSPGPCGA** |  |  |  |  |  |  |  | 31.21 |  |  | **ADPIPSGRSPGPCGA** | **LINC00273** |
| **69** | **GBM-D** | **MKDCQLRKQQNENVS** |  |  |  |  |  |  |  |  | 41.12 |  | **MKDCQLRRQQNENVS** | **SLFN12L** |
| **70** | **GBM-D** | **VKRNPPKTAKVSEPG** | 246.07 | 1275.82 | 43.49 | 168.92 |  |  |  |  | 9.24 |  | **VKRNPPKKAKVSEPG** | **HOXB1** |
| **71** | **GBM-D** | **GSGPSCRRWEKKLAS** | 376.6 |  | 106.84 |  |  |  |  |  |  |  | **GSGPSCRQWEKKLAS** | **PIK3R5** |
| **72** | **GBM-D** | **DMYGTGQESLYS** |  | 472.45 |  | 126.31 |  |  |  |  |  |  | **DMYGTGQQSLYS** | **CDH7** |
| **73** | **GBM-D** | **QSYKNDFNAEYSEYR** |  | 385.13 |  |  |  |  |  |  |  |  | **QSYKNDFSAEYSEYR** | **ELL** |
| **74** | **GBM-D** | **ARKAKYNVHATVRYQ** |  | 376.14 |  | 81.38 |  | 314.34 |  |  |  |  | **ARKAKYNLHATVRYQ** | **NCAN** |
| **75** | **GBM-D** | **MRVMKFSVSPVVRVA** |  |  |  |  |  |  | 258.99 |  |  |  | **MRVMKFSISPVVRVA** | **EEF2** |
| **76** | **GBM-D** | **YAPCGDLSGMLQERG** |  |  |  |  |  |  |  |  |  | 23.08 | **YAPCGDLNGMLQERG** | **SBK3** |
| **77** | **GBM-D** | **GQLAVSKRLALEVTV** |  |  |  |  |  |  | 304.12 |  | 39.39 |  | **GQLAVSKCLALEVTV** | **SIRPG** |
| **78** | **GBM-D** | **QRAAAIARQKAEIAA** |  | 889.28 |  |  |  | 24.41 |  |  | 112.28 |  | **QRAAAIACQKAEIAA** | **JPH2** |
| **79** | **GBM-D** | **ISPSRAARQLMERTQ** | 606.87 |  |  |  |  |  |  |  |  |  | **ISPSRAACQLMERTQ** | **ELMO2** |
| **80** | **GBM-D** | **FARKLKDVHETLGFP** |  | 410.66 |  |  |  |  |  |  |  |  | **FARKLKDIHETLGFP** | **TTN** |
| **81** | **GBM-D** | **EPDNIKYVISEEKGS** |  | 361.16 |  | 143.5 |  |  |  |  |  |  | **EPDNIKYMISEEKGS** | **TTN** |
| **82** | **GBM-D** | **DNHCEQLRVKIRKLK** | >1864.49 | >1864.49 | 194.67 | 85.46 |  |  |  |  |  |  | **DNHCEQLGVKIRKLK** | **ANKRD36C** |
| **83** | **GBM-D** | **LTELKDNHCEQLRVK** | 495.5 | 561.95 |  | 117.31 |  | 24.41 | 1759.72 |  |  |  | **LTELKDNLCEQLRVK** | **ANKRD36C** |
| **84** | **GBM-D** | **DFSVIIMAYVSENIK** | 669.42 |  | 401.29 |  |  |  |  |  |  |  | **DFSVIIMVYVSENIK** | **SCN5A** |
| **85** | **GBM-D** | **GKGVMLAVSQGRVQT** | 328.34 | 676.71 | 213.7 |  |  |  |  | 702.12 |  |  | **GKGVMLAISQGRVQT** | **TENM3** |
| **86** | **GBM-D** | **SRELCPGRWRAGPWS** | 467.9 |  | 170.5 |  |  |  | 1023.03 |  |  |  | **SRELCPGHWRAGPWS** | **ADAMTS2** |
| **87** | **GBM-D** | **WDLTDALRLAALSIE** |  |  |  |  |  |  | 423.55 |  |  |  | **WDLTDALWLAALSIE** | **LAMA4** |
| **88** | **GBM-D** | **SHLIAASNCHSLELQ** | 993.08 |  |  |  |  |  |  |  |  |  | **SHLIAASSCHSLELQ** | **CAPN11** |
| **89** | **GBM-D** | **EDVKWPPTLQPPTLR** |  |  |  |  |  |  | 682.55 |  |  |  | **EDVKWPPPTLQPPTL** | **IRF5** |
| **90** | **GBM-D** | **NGMEWNGMEWNRIES** | 343.39 |  | 61.44 |  |  |  |  |  |  |  | **NGMEWNGIEWNRIES** | **PCSK5** |
| **91** | **GBM-D** | **AATSHPKPTTGHIKP** | 307.31 | 1000.95 | 143.79 |  |  |  |  |  |  |  | **AATSHPKHIKPATSH** | **GPR50** |
| **GBM-E** | |  |  |  |  |  |  |  |  |  |  |  |  |  |
| **92** | **GBM-E** | **ALYDICSKTLKLPTP** | 193.09 | 570.95 | 269.11 | 225.35 | 35.32 | 62.77 | 64.68 |  |  | 22.69 | **ALYDICSRTLKLPTP** | **TUBB8** |
| **93** | **GBM-E** | **SSGGCCGSSSGGCCS** |  | 63.38 |  | 44.81 |  |  |  |  |  |  | **SSGGCCGSSSGGCCS** | **LCE1F** |
| **94** | **GBM-E** | **REQEEKMWRQEEKIR** |  |  |  |  |  |  |  |  | 126.84 | 85.39 | **REQEEKMRRQEEKIR** | **NCKAP1L** |
| **95** | **GBM-E** | **REDAGAGGEDVGAGG** |  | 88.74 |  | 12.26 |  |  |  |  |  |  | **REDAGAGEEDVGAGG** | **GOLGA6L1** |
| **96** | **GBM-E** | **IREQEEMLREQEAQR** | 105.48 | 754.23 |  | 101.35 | 55.96 | 7.17 |  |  |  | 35.73 | **IREQEEMiREQEAQR** | **GOLGA6L2** |
| **97** | **GBM-E** | **PPTWSGRRAPGDRDN** |  | 270.84 |  | 175.5 |  | 92.98 |  |  |  |  | **PPTWSGRHAPGDRDN** | **LOC645752** |
| **98** | **GBM-E** | **QFLIPTSLSVSSNSV** |  | 30.48 |  | 99.34 |  | 10.73 |  |  |  |  | **QFLIPTSFSVSSNSV** | **DSPP** |
